# The Tumor Microenvironment of Pancreatic Cancer

**DOI:** 10.3390/cancers12103076

**Published:** 2020-10-21

**Authors:** Eva Karamitopoulou

**Affiliations:** Pancreatic Cancer Research Group, Institute of Pathology University of Bern, CH-3008 Bern, Switzerland; eva.diamantis@pathology.unibe.ch

Pancreatic ductal adenocarcinoma (PDAC) has a dismal prognosis along with rising incidence rates and will be responsible for many cancer deaths in the future [1,2]. Most patients present with metastatic and/or locally advanced, non-resectable disease and can only be treated with palliative chemotherapy [1,2,3]. PDAC patients with (borderline) resectable disease can profit from an oncologic resection [4,5]; however, a significant number of these patients will eventually develop recurrent disease a few months after resection [6]. The use of neoadjuvant chemoradiation (with or without systemic chemotherapy), which can improve the resectability of PDAC, as well as the administration of postoperative adjuvant chemotherapy with more modern regimens such as FOLFIRINOX (combination of Fluoruracil, Folinsäure (Leucovorin), Irinotecan and Oxaliplatin) and gemcitabine/nabpaclitaxacel, have contributed to further, modest improvements in survival [7,8,9,10]. However, in order to substantially improve the survival rates of PDAC patients, we need new, more individualized clinical management strategies, including a more accurate prognostic/predictive patient classification.

Recently, genomic and transcriptomic profiling has allowed the characterization of distinct molecular PDAC subtypes with unique genetic signatures, deepening our knowledge of the intrinsic mechanisms of PDAC progression [11,12,13,14]. However, the translation of these findings into clinical practice has so far been very limited. One of the reasons for this is the relative lack of actionable targets as well as the widespread intra-tumoral heterogeneity (ITH) present in many solid tumors including PDAC and known to correlate with tumor progression and poor outcomes [15,16,17,18,19]. In this context, the evolutionary diversity within the tumor is driving the emergence of aggressive subclones, especially under pressure from therapeutic agents.

During PDAC evolution, the complexity and the dynamic interactions between tumor and immune cells within the tumor microenvironment (TME) play important roles in the pathogenesis and progression of the disease [20]. On the one hand, the tumor cells often display oncogenic mutations that help them evade anti-tumor immunity, while on the other hand, the TME of PDAC can also influence the local immune response [20,21,22,23]. Thus, the immunoarchitectural characteristics of the TME interact and cooperate with the tumor cells in a dynamic way to affect tumor progression. Therefore, when it comes to PDAC, analysis of the TME has to be taken into account as well. 

The TME is a very complex ecosystem in which several factors, such as immune cells of the innate and adaptive immunity, cytokines and other immunoregulatory molecules, extracellular matrix as well as stromal fibroblasts, are involved, contributing to the development of a frequently immunosuppressive, highly hypoxic and desmoplastic tumor, resistant to all kinds of therapy, including chemotherapy, targeted therapy and immunotherapy [20]. For example, PDAC is known to be associated with a rich, strongly desmoplastic stroma comprising cancer-associated fibroblasts (CAFs), pancreatic stellate cells and extracellular matrix, resulting in a tumor that is highly hypoxic and hypovascular [24]. Moreover, the TME of PDAC is frequently characterized by a low number of tumor infiltrating lymphocytes (TILs) and a high number of T regulatory cells (Tregs) and myeloid-derived suppressor cells (MDSCs), which serve to decrease the tumor-specific immune response [25]. This immunosuppressive and desmoplastic TME is thought to help tumor cells evade anti-tumor immune response by impeding the access of T cells and precluding immune cells from the tumor cells, a phenomenon known as immune privilege [26]. It is therefore important for TME factors to also be considered in the view of overcoming therapy resistance [12]. Some encouraging evidence comes, however, from studies on long-term survivors of PDAC. These recent studies have shown that the neoantigen quality (rather than quantity) can modulate immunogenicity in PDAC and have reported that both a high neoantigen number and an abundant CD8+ T-cell infiltrate was present in the tumors of long-term survivors, implying that the neoantigenic repertoire has been evolutionary selected in this cohort [27]. This underlines the importance of understanding and expanding our knowledge of the spectrum of tumor-immune interactions. 

This is especially important regarding immunotherapy. Early clinical trials with checkpoint inhibitors, including monotherapy with monoclonal antibodies that block PD-L1 from binding its ligand, have yielded rather disappointing results in pancreatic cancer, apart from a few cases with microsatellite instability [28,29]. Thus, studies to detect further potential predictive biomarkers correlated to immunotherapy outcomes are necessary. Based on immunopathology and gene expression approaches, the TME of most cancers can be broadly characterized as either T cell inflamed (“hot”) or non-T cell inflamed (“cold”), depending on the frequency, composition and spatial organization of tumor-infiltrating lymphocytes (TILs) and immunomodulatory molecules [30,31] (Figure 1A,B). Generally, T cell inflamed tumors exhibit improved responses to immunotherapies [31]. Moreover, histopathologic examination of the TME can also reveal critical tumor-intrinsic characteristics. For example, biologically aggressive PDACs display increased numbers of dissociative growing tumor cells exhibiting partial epithelial–mesenchymal transition (EMT)-features, termed tumor buds. Tumor budding is an independent adverse prognostic factor in PDAC and is associated with an immunosuppressive TME [32,33].

Thus, insights into the different landscapes of PDAC would promote our understanding of the biology behind the tumor–immune interactions and would support a more accurate prognostic and predictive stratification of patients for more efficient clinical management. Moreover, integrative approaches, which take into account intrinsic tumor features, such as morphology and genetic changes in the tumor cells as well as extrinsic features such as the immune landscape of the TME, would provide a more efficient tool to tackle this recalcitrant disease, towards a more individualized clinical management of PDAC patients. As newer treatment regimens become available, both in the adjuvant and in the neoadjuvant settings, including immunotherapy [30,34], these approaches would help selecting patient subsets for appropriate treatment modalities. 

## Figures and Tables

**Figure 1:**
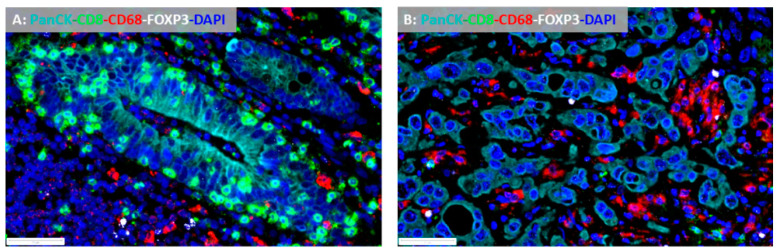
(**A**): Pancreatic cancer from a long-term survivor (i.e., overall survival >60 months) with a «hot» tumor microenvironment, dominated by CD8+ cytotoxic tumor infiltrating lymphocytes (multiplex immunofluorescence ×400); (**B**): pancreatic cancer from a short-term survivor (overall survival <12 months) with an immunosuppressive tumor microenvironment poor in CD68+ tumor infiltrating lymphocytes and dominated by CD68+ tumor associated macrophages and FOXP3+ T regulatory cells (multiplex immunofluorescence ×400).

## References

[1] Kamisawa T., Wood L.D., Itoi T., Takaori K. (2016). Pancreatic cancer. Lancet.

[2] Rahib L., Smith B.D., Aizenberg R., Rosenzweig A.B., Fleshman J.M., Matrisian L.M. (2014). Projecting cancer incidence and deaths to 2030: The unexpected burden of thyroid, liver, and pancreas cancers in the United States. Cancer Res..

[3] Ryan D.P., Hong T.S., Bardeesy N. (2014). Pancreatic adenocarcinoma. N. Engl. J. Med..

[4] Serrano P.E., Cleary S.P., Dhani N., Kim P.T., Greig P.D., Leung K., Moulton C.A., Gallinger S., Wei A.C. (2015). Improved long-term outcomes after resection of pancreatic adenocarcinoma: A comparison between two time periods. Ann. Surg. Oncol..

[5] Bahra M., Pratschke J., Klein F., Neuhaus P., Boas-Knoop S., Puhl G., Denecke T., Pullankavumkal J.R., Sinn M., Riess H. (2015). Cytoreductive Surgery for Pancreatic Cancer Improves Overall Outcome of Gemcitabine-Based Chemotherapy. Pancreas.

[6] Sakamoto H., Attiyeh M.A., Gerold J.M., Makohon-Moore A.P., Hayashi A., Hong J., Kappagantula R., Zhang L., Melchor J.P., Reiter J.G. (2020). The evolutionary origins of recurrent pancreatic cancer. Cancer Discov..

[7] Conroy T., Hammel P., Hebbar M., Ben Abdelghani M., Wie A.C., Raoul J.L., Choné L., Francois E., Artru P., Biagi J.J. (2018). FOLFIRINOX or Gemcitabine as Adjuvant Therapy for Pancreatic Cancer. N. Engl. J. Med..

[8] Oettle H., Neuhaus P., Hochhaus A., Hartmann J.T., Gellert K., Ridwelski K., Niedergethmann M., Zülke C., Fahlke J., Arning M.B. (2013). Adjuvant chemotherapy with gemcitabine and long-term outcomes among patients with resected pancreatic cancer: The CONKO-001 randomized trial. JAMA.

[9] Hoff D.D., Von Ervin T., Arena F.P., Chiorean E.G., Infante J., Moore M., Seay T., Tjulandin S.A., Ma W.W., Saleh M.N. (2013). Increased survival in pancreatic cancer with nab-paclitaxel plus gemcitabine. N. Engl. J. Med..

[10] Trinh K.V., Fischer D.A., Gardner T.B., Smith K.D. (2020). Outcomes of Neoadjuvant Chemoradiation with and Without Systemic Chemotherapy in Resectable and Borderline Resectable Pancreatic Adenocarcinoma. Front. Oncol..

[11] Moffitt R.A., Marayati R., Flate E.L., Volmar K.E., Herrera Loeza S.G., Hoadley K.A., Rashid N.U., Williams L.A., Eaton S.C., Chung A.H. (2015). Virtual microdissection identifies distinct tumor- and stroma-specific subtypes of pancreatic ductal adenocarcinoma. Nat. Genet..

[12] Collisson E.A., Sadanandam A., Olson P., Gibb W.J., Truitt M., Gu S., Cooc J., Weinkle J., Kim G.E., Jakkula L. (2011). Subtypes of pancreatic ductal adenocarcinoma and their differing responses to therapy. Nat. Med..

[13] Bailey P., Chang D.K., Nones K., Johns A.L., Patch A.M., Gingras M.C., Christ A.N., Bruxner T.J., Quinn M.C., Nourse C. (2016). Genomic analyses identify molecular subtypes of pancreatic cancer. Nature.

[14] Puleo F., Nicolle R., Blum Y., Cros J., Marisa L., Demeter P., Quertinmont E., Svrcek M., Elarouci N., Iovanna J. (2018). Stratification of Pancreatic Ductal Adenocarcinomas Based on Tumor and Microenvironment Features. Gastroenterology.

[15] Greaves M., Maley C.C. (2012). Clonal evolution in cancer. Nature.

[16] Andor N., Graham T.A., Jansen M., Xia L.C., Aktipis C.A., Petritsch C., Ji H.P., Maley C.C. (2016). Pan-cancer analysis of the extent and consequences of intratumor heterogeneity. Nat. Med..

[17] McGranahan N., Swanton C. (2017). Clonal Heterogeneity and Tumor Evolution: Past, Present, and the Future. Cell.

[18] Dagogo-Jack I., Shaw A.T. (2018). Tumour heterogeneity and resistance to cancer therapies. Nat. Rev. Clin. Oncol..

[19] Reiter J.G., Baretti M., Gerold J.M., Makohon-Moore A.P., Daud A., Iacobuzio-Donahue C.A., Azad N.S., Kinzler K.W., Nowak M.A., Vogelstein B. (2019). An analysis of genetic heterogeneity in untreated cancers. Nat. Rev. Cancer.

[20] Dougan S.K. (2017). The pancreatic cancer microenvironment. Cancer J..

[21] Binnewies M., Roberts E.W., Kersten K., Chan V., Fearon D.F., Merad M., Coussens L.M., Gabrilovich D.I., Ostrand-Rosenberg S., Hedrick C.C. (2018). Understanding the tumor immune microenvironment (TIME) for effective therapy. Nat. Med..

[22] Karamitopoulou E. (2019). Tumour microenvironment of pancreatic cancer: Immune landscape is dictated by molecular and histopathological features. Br. J. Cancer.

[23] Wartenberg M., Cibin S., Zlobec I., Vassella E., Eppenberger-Castori S., Terracciano L., Eichmann M.D., Worni M., Gloor B., Perren A. (2018). Integrated Genomic and Immunophenotypic Classification of Pancreatic Cancer Reveals Three Distinct Subtypes with Prognostic/Predictive Significance. Clin. Cancer Res..

[24] Thomas D., Radhakrishnan P. (2019). Tumor-stromal crosstalk in pancreatic cancer and tissue fibrosis. Mol. Cancer.

[25] Martinez-Bosch N., Vinaixa J., Navarro P. (2018). Immune Evasion in Pancreatic Cancer: From Mechanisms to Therapy. Cancers.

[26] Johnson B.A., Yarchoan M., Lee V., Laheru D.A., Jaffee E.M. (2017). Strategies for increasing pancreatic tumor immunogenicity. Clin. Cancer Res..

[27] Balachandran V.P., Luksza M., Zhao J.N., Makarov V., Moral J.A., Remark R., Herbst B., Askan G., Bhanot U., Senbabaoglu Y. (2017). Identification of unique neoantigen qualities in long-term survivors of pancreatic cancer. Nature.

[28] Sodergren M.H., Mangal N., Wasan H., Sadanandam A., Balachandran V.P., Jiao L.R., Habib N. (2020). Immunological combination treatment holds the key to improving survival in pancreatic cancer. J. Cancer Res. Clin. Oncol..

[29] Eso Y., Shimizu T., Takeda H., Takai A., Marusawa H. (2020). Microsatellite instability and immune checkpoint inhibitors: Toward precision medicine against gastrointestinal and hepatobiliary cancers. J. Gastroenterol..

[30] Galon J., Bruni D. (2019). Approaches to treat immune hot, altered and cold tumours with combination immunotherapies. Nat. Rev. Drug Discov..

[31] Trujillo J.A., Sweis R.F., Bao R., Luke J.J. (2018). T Cell–Inflamed versus Non-T Cell–Inflamed Tumors: A Conceptual Framework for Cancer Immunotherapy Drug Development and Combination Therapy Selection. Cancer Immunol. Res..

[32] Karamitopoulou E., Zlobec I., Born D., Kondi-Pafiti A., Lykoudis P., Mellou A., Gennatas K., Gloor B., Lugli A. (2013). Tumour budding is a strong and independent prognostic factor in pancreatic cancer. Eur. J. Cancer.

[33] Lohneis P., Sinn M., Klein F., Bischoff S., Striefler J.K., Wislocka L., Sinn B.V., Pelzer U., Oettle H., Riess H. (2018). Tumour buds determine prognosis in resected pancreatic ductal adenocarcinoma. Br. J. Cancer.

[34] Whiteside T.L., Demaria S., Rodriguez-Ruiz M.E., Zarour H.M., Melero I. (2016). Emerging opportunities and challenges in cancer immunotherapy. Clin. Cancer Res..

